# Accelerometric outcomes of motor function related to clinical evaluations and muscle involvement in dystrophic dogs

**DOI:** 10.1371/journal.pone.0208415

**Published:** 2018-12-11

**Authors:** Mutsuki Kuraoka, Yuko Nitahara-Kasahara, Hisateru Tachimori, Naohiro Kato, Hiroyuki Shibasaki, Akihiko Shin, Yoshitsugu Aoki, En Kimura, Shin’ichi Takeda

**Affiliations:** 1 Department of Molecular Therapy, National Institute of Neuroscience, National Center of Neurology and Psychiatry, Kodaira, Tokyo, Japan; 2 Laboratory of Experimental Animal Science, Nippon Veterinary and Life Science University, Musashino, Tokyo, Japan; 3 Department of Biochemistry and Molecular Biology, Nippon Medical School, Bunkyo-ku, Tokyo, Japan; 4 Department of Clinical Epidemiology, Translational Medical Center, National Center of Neurology and Psychiatry, Kodaira, Tokyo, Japan; 5 Research Center for Medical and Health Data Science, The Institute of Statistical Mathematics, Tachikawa, Tokyo, Japan; 6 Department of Gene Regulation, Faculty of Pharmaceutical Sciences, Tokyo University of Science, Noda, Chiba, Japan; 7 School of Medicine, Shinshu University, Matsumoto, Nagano, Japan; 8 Translational Medical Center, National Center of Neurology and Psychiatry, Kodaira, Tokyo, Japan; University of Minnesota Medical Center, UNITED STATES

## Abstract

Duchenne muscular dystrophy (DMD) is an X-linked muscle disorder characterized by primary muscle degeneration. Patients with DMD reveal progressive muscle weakness leading to ambulatory dysfunction. Novel outcome measures are needed for more sensitive evaluation of therapeutic effects in clinical trials. Multiple parameters of acceleration and angular velocity are used as efficient indicators to quantify the motion of subjects, and these parameters have been recently applied for evaluation of motor function in DMD. In the present study, we evaluated gait in a dystrophic dog model, CXMD_J_, by measuring three-axial acceleration and angular velocity over the course of months. Hybrid sensors were placed on the dorsal thoracic and lumbar regions of dogs to detect a wide range of acceleration (±8 G) and angular velocity (±1000 degrees per second). Multiple parameters showed lower values in dystrophic dogs compared to wild-type (WT) dogs, and declined over the course of months. Acceleration magnitude (*AM*) at the thoracic region in dystrophic dogs was prominently lower compared with WT dogs, even at the age of 2 months, the onset of muscle weakness, whereas *AM* at the lumbar region drastically declined throughout the disease course. The angular velocity index in the vertical direction in the lumbar region increased in dystrophic dogs, suggesting waddling at the girdle. These parameters also accordingly decreased with exacerbation of clinical manifestations and a decrease in spontaneous locomotor activity. The *AM* of dystrophic dogs was analyzed with magnetic resonance imaging to look for a correlation with crus muscle involvement. Results showed that acceleration and angular velocity are multifaceted kinematic indices that can be applied to assess outcomes in clinical trials for hereditary neuromuscular disorders including DMD.

## Introduction

Duchenne muscular dystrophy (DMD) is an X-linked disorder of muscle characterized by primary muscle degeneration [1]. Its prevalence in the population is estimated to be 1 in 5000 male newborns. A mutation in *DMD* results in the absence of dystrophin, a structural protein in muscle fibers, leading to fragility of muscle fibers following contractive force [2]. Histologic features of DMD are muscle fiber degeneration with secondary cellular inflammation, ineffective muscle fiber regeneration, and eventually fibrosis and adiposis. Hence, DMD patients have muscle weakness, leading to loss of ambulation and early death from respiratory or cardiac failure.

Therapeutic strategies for DMD, such as gene and cell therapy and pharmaceuticals, have been explored in human trials [3–6]. With the development of new therapies, sensitive outcome measures are needed to capture disease progression and monitor treatment effects. The 6-minute walking test, which measures the distance walked in 6 minutes, is a primary outcome measure of motor function in DMD [7, 8], but this test is not sufficiently sensitive to measure disease progression in younger boys [9].

Miniature body-fixed motion sensors have been recently developed, and thus accelerometry is now an efficient and sensitive method to quantify performance in validated tasks and daily living activities [10–12]. Physical activities involving both ambulant and non-ambulant conditions in DMD have been assessed in patients by measuring acceleration parameters that indicate movement and orientation of the body and upper limbs [13–16]. Accelerometry for informal tasks such as walking is used to monitor disease progression as well as corticosteroid treatment effects [17, 18]. Accelerometry is also more practical for capturing motion when combined with different types of information such as angular velocity [12, 16, 17].

Magnetic resonance imaging (MRI) has been used increasingly in DMD studies [19, 20]. T2-weighted imaging and two-point Dixon methods capture muscle conditions that involve necrosis, inflammation, and non-contractile tissue infiltration [21–23]. Quantitative MRI signals of the thigh and crus muscles are often highly correlated with muscle weakness and physical activities [22–24]. Dystrophic muscles also show changes in myofiber-type populations, that is, progressive depletion of fast myofibers and a shift towards a predominance of slow myofiber populations [25–27]. This observation has led to the hypothesis that motor function in DMD is primarily related to a decrease in fast myofibers.

In the present study, the gait in a dystrophic model of beagle dogs, canine X-linked muscular dystrophy in Japan (CXMD_J_), was longitudinally evaluated by measuring three-axial acceleration and angular velocity. These parameters in dystrophic dogs were also assessed the relationship to clinical severity and MRI signals of fast and slow muscles in the lower legs. In previous studies, gait abnormalities in golden retriever muscular dystrophy (GRMD) were observed with an accelerometer, which detects acceleration within a range of ±2 G [28–30]. To evaluate gait, including the maximal driving force in dystrophic and wild-type (WT) dogs, we used a hybrid sensor to measure acceleration and angular velocity with a wider range of ±8 G and ±1000 degrees per second (dps), respectively. We found that multiple parameters of acceleration and angular velocity declined according to disease severity and as the disease progressed, and that these parameters varied between the dorsal thoracic and lumbar regions.

## Materials and methods

### Animals

A CXMD_J_ dog colony was established by insemination of beagles with the sperm of GRMD dogs [31]. CXMD_J_ dystrophic dogs lack dystrophin in the muscle tissue and have dystrophic phenotypes, as observed in GRMD and in human DMD [32]. The present study was approved by the Ethics Committee for the Treatment of Laboratory Middle-sized Animals of the National Institute of Neuroscience (Approval No.: 27–02, 28–02). All dogs were cared for and treated in accordance with the guidelines of the Committee. Motor function and clinical tests were performed in five CXMD_J_ dystrophic and six WT dogs in the fourth to eighth generations from the first artificial insemination from 2 to 12 months of age. MRI was performed at the age of 1 year. Subject information is grouped by littermates (Table 1).

**Table 1:** Subject information.

Dog ID	Phenotype	Gender
13103FN	WT	F
13102MA	Dys	M
13301MN	WT	M
13303MA	Dys	M
13401MA	Dys	M
13402FN	WT	F
13802MA	Dys	M
13804MN	WT	M
13805MN	WT	M
14102MA	Dys	M
14103MN	WT	M

Subjects are listed in order of littermates. WT, wild type; Dys, dystrophic; F, female; M, male.

### Motor function test

**Fig 1:**
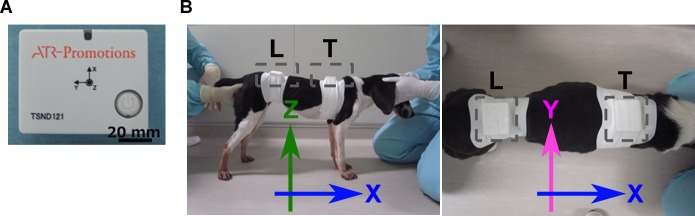
Portable sensors and areas where subjects wore sensors. (A) A portable sensor TSND121 for measurement of three-axial acceleration and angular velocity. (B) Sensors were worn on the dorsal thoracic and lumbar regions. The three axes are the X-axis for the caudal-cranial direction, the Y-axis for the medial-lateral direction, and the Z-axis for the ventral-dorsal direction. T, thoracic; L, lumbar.

Motor function of dystrophic and WT dogs was evaluated by assessing multiple parameters derived from acceleration and angular velocity during ambulation. Portable wireless hybrid sensors TSND121 (ATR-Promotions, Inc., Soraku-gun, Kyoto, Japan) (Fig 1A), which were sized 46 × 37 × 12 mm and weighed 22 g, were used to measure three-axial acceleration and angular velocity. Sensors were affixed with a stretchy sticky bandage (3M Company, St. Paul, MN, USA) and worn around the sixth thoracic and seventh lumbar vertebrae of subjects to assess the dorsal thoracic and lumbar regions, respectively (Fig 1B). The three axes were the X-axis (caudal-cranial), Y-axis (medial-lateral), and Z-axis (ventral-dorsal). All data were recorded using SDRecorderT software version 1.3.3 (ATR-Promotions, Inc.) in a computer from trials in which a dog ran down a hallway (15 m × 4 times) [33, 34]. If a dog sat down before completing the 15-m distance due to fatigue, the trial was stopped. The specific acceleration vector indicates the instantaneous inertial acceleration for each axis (*Ax*, *Ay*, *Az*) and is expressed in G-force (1 G = 9.81 m/sec^2^). The instantaneous angular velocity vector indicates the instantaneous rotation of the trunk (*Gx*, *Gy*, *Gz*) and is expressed in dps. Three-axial acceleration (± 8G) and angular velocity (± 1000 dps) were sampled at 0.24 mG and 0.03 dps per 20 milliseconds, respectively, with analog-digital acquisition. Data from the thoracic region were incomplete in subject ID 13103FN at the age of 2, 3, 4, 5, and 11 months, 13102MA at the age of 2, 3, 4, and 5 months, and 13303MA and 13301MN at the age of 2 and 3 months.

### Signal processing and variable computation

Raw data for acceleration and angular velocity were extracted between start and goal on a time line, using SDLabelerT software version 1.30 (ATR-Promotions, Inc.). Using SensorDataAnalyzer software version 1.0.0 (ATR-Promotions, Inc.), all raw data for acceleration were corrected by removal of offset (gravity) as a component of direct current for 10 seconds while the dog was in a quadrupedal standing position. In the trials of subject ID 13103FN and 13102MA at the age of 2, 3, 4, and 5 months and 13301MN at the age of 5 months, offset was sampled for 2–5 seconds. The instantaneous vectors of acceleration (*Ax*, *Ay*, *Az*) and angular velocity (*Gx*, *Gy*, *Gz*) were calculated as the average of absolute values of each axis. Acceleration magnitude (*AM*) was also calculated from the three acceleration vectors (*Ax*, *Ay*, *Az*) as the square root of the sum of the three-axial values (*AM* = √*Ax*^2^ + *Ay*^2^ + *Az*^2^) [35], and was averaged for each trial. The relative components of the *AM* along the three axes (%) were calculated by dividing the absolute values of each axis by the *AM* [29], and these components that were averaged in each trial were calculated as acceleration ratios (*Ax* ratio, *AY* ratio, *AZ* ratio).

In time series analysis, we estimated trends of multiple parameters of acceleration and angular velocity in dystrophic and WT dogs. For both groups, we assumed the following local level models. In these models, i and t are indices of “dog” and “month,” respectively. For acceleration or angular velocity, we assumed the following model:

$$\log\left(y_{it}\right)∼N(m_{t},\sigma_{0}^{2}).$$

For the acceleration ratio, we assumed the following model:

$$\log\left(\frac{r_{it}}{100-r_{it}}\right)∼N(m_{t},\sigma_{0}^{2}),$$

where y_it_ is acceleration or angular velocity, r_it_ is the acceleration ratio, and m_t_ is the trend. For m_t_ of the above two models, we assumed the following model:

$$m_{t}∼N(m_{t-1},{\sigma_{1}}^{2}).$$

Time series of multiple parameters were compared between dystrophic and WT dogs at thoracic and lumbar regions.

### Clinical manifestations

Clinical evaluation of dystrophic dogs was performed as described in our previous reports [32–34, 36, 37]. Briefly, we evaluated gait and mobility abnormalities, limb and temporal muscle atrophy, drooling, macroglossia, dysphagia, and abnormal sitting posture as clinical signs. The severity of each sign was classified as a score of 1 to 5 (grade 1, none; grade 5, severe) according to a grading scale for CXMD_J_ [36]. Data for subject ID 13303MA at the age of 4 months and 13401MA at the age of 2 months were absent.

### Spontaneous locomotor activity analysis

Spontaneous locomotor activity of dystrophic and WT dogs was analyzed as described in a previous report [34, 37] with modifications. All dogs were kept in a test cage (W960×H1565×D1652 mm) and monitored using an infrared monitoring system (Supermex, MUROMACHI KIKAI, Tokyo, Japan). The system consists of a sensor mounted above the cage that detects changes in heat across multiple zones of the cage using an array of Fresnel lenses. In this way, the system monitors and counts all spontaneous movements, both vertical and horizontal, every 10 min over 5 days. All summed counts were automatically recorded. Summed counts between 07:00 and 09:00 were extracted to remove influence of animal keepers feeding or cleaning cages during the lighting period. Data of subject ID 13401MA at the age of 2 and 3 months were absent.

### MRI

All anesthetized dogs were evaluated for muscle involvement using MRI, as previously described [33]. Anesthesia in the dystrophic and WT dogs was induced by intravenous injection of 20 mg/kg thiopental sodium (Ravonal, Mitsubishi Tanabe Pharma, Osaka, Japan) and maintained by inhalation of isoflurane (Isoflu, DS Pharma Animal Health Co., Osaka, Japan). We examined the crus muscles of the lower limbs with a superconducting 3.0-Tesla MRI device (MAGNETOM Trio; Siemens Medical Solutions, Erlanger, Germany) with an 18-cm diameter/18-cm length human extremity coil. The acquisition parameters for T2-weighted imaging were TR/TE = 4,000/89 milliseconds, slice thickness = 4 mm, slice gap = 4 mm, field of view = 128 × 128 mm, matrix size = 256 × 256, and number of acquisitions = 9 during fast spin echo.

Quantitative analysis of the images was performed using Syngo MR2004A software (Siemens Medical Solutions), as previously reported [21]. Briefly, regions of interest (ROIs) were selected to avoid flow artifacts and large vessels. Two or three ROIs were manually captured in both right and left muscles of serial images and anatomically conformed as described previously [38]. Signal intensities were measured in these ROIs. Signal-to-noise ratios (SNRs) of each ROI were calculated with the equation: SNR = signal intensity/SD_air_, where SD_air_ was the standard deviation (SD) of the background noise [39]. The average SNR (Ave SNR) was calculated with equation: Ave SNR = {(Σ(SNR_i,Right_ × Pixel_i,Right_)+Σ(SNR_i,Left_ × Pixel_i,Left_)}/Pixel_Total_ (i = 1,2 or 1,2,3). Crus muscles were grouped into fast and slow muscles based on the population of myofibers that expressed the myosin heavy chain isoforms [40–43].

### Statistical analysis

Multiple parameters of acceleration and angular velocity were analyzed for their relationship with clinical manifestations or spontaneous locomotor activity. The relationship was estimated with the following local level model:

$$\log\left(y_{it}\right)∼N(m_{t}+\beta x_{it},\sigma_{0}^{2}),$$

$$\log\left(\frac{r_{it}}{100-r_{it}}\right)∼N(m_{t}+\beta x_{it},\sigma_{0}^{2}),$$

where y_it_ is acceleration or angular velocity, *x*_*it*_ is the clinical manifestation or spontaneous locomotor activity, and m_t_ is the trend.

For comparison of Ave SNRs of crus muscles on T2-weighted images, the median values were compared between dystrophic and WT dogs using the Mann-Whitney U test. Pearson’s correlation test was used to test relationships among multiple parameters and Ave SNRs of crus muscles. The statistical significance level was set at 5%. The local level models were estimated with OpenBUGS version 3.2.3, MCMC software, which is available at http://www.openbugs.net/. The Mann-Whitney U test was performed with StatView-J software version 5.0 (SAS Institute Inc., Cary, NC). Pearson’s correlation test was performed with R 3.4.0, which is available at https://www.R-project.org/.

## Results

### Acceleration parameters in dystrophic and WT dogs

**Fig 2:**
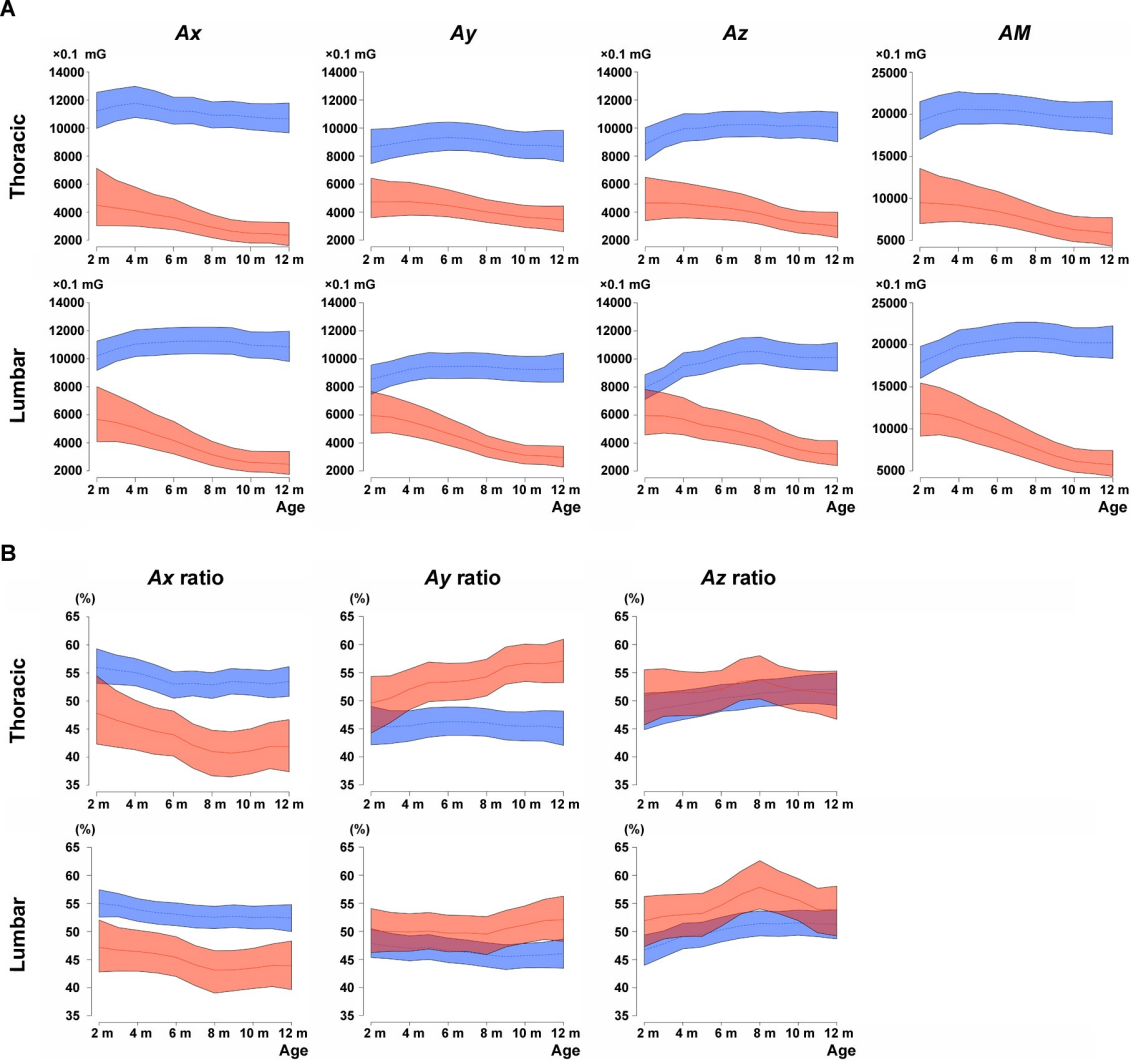
Acceleration parameters of wild-type and dystrophic dogs at different ages. Averages of (A) absolute values of three-axial acceleration (*Ax*, *Ay*, and *Az*) and acceleration magnitude (*AM*) and (B) three-axial acceleration ratios (*Ax* ratio, *Ay* ratio, and *Az* ratio). Lines and shaded surrounding areas in wild-type (WT, blue) and dystrophic dogs (red) indicate estimations and 95% credible intervals of the trends in acceleration parameters, respectively.

We evaluated the gait of dystrophic and WT dogs using acceleration parameters during running for 15 m. WT dogs mostly ran with a gallop in all trials. Dystrophic dogs showed a bunny hop that co-instantaneously drove both hindlimbs at the stance and swing phases during the gallop. Dystrophic dogs changed their gait pattern to a trot or walk according to the severity (S1 Table). Acceleration waves for the X, Y, and Z axes are shown for dystrophic and WT dogs in S1 Fig. All waves of dystrophic dogs showed lower and broader amplitudes than those of WT dogs during the gallop and were smaller during the trot. The instantaneous vectors of acceleration (*Ax*, *Ay*, *Az*) were compared between dystrophic and WT dogs at different ages (Fig 2A). All three-axial vectors of dystrophic dogs were lower than those of WT dogs both at the dorsal thoracic and lumbar regions and were progressively attenuated over the course of months. *AM*s in WT dogs were increased and peaked at the age of 4 and 7–8 months in thoracic and lumbar regions, respectively, and *AM*s in dystrophic dogs were lower compared with those of WT dogs (Fig 2A). At the age of 2 months, which is the onset of muscle weakness in dystrophic dogs [32], *AM*s in dystrophic dogs were already lower compared with WT dogs, especially in the thoracic region. *AM*s in dystrophic dogs were also attenuated over the course of months and were especially severe at the lumbar region.

We next analyzed three-axial acceleration ratios (*Ax* ratio, *Ay* ratio, *Az* ratio) to detect three-axial bias for whole acceleration (Fig 2B). *Ax* ratios in the dorsal thoracic and lumbar regions were lower in dystrophic dogs compared with WT dogs. The *Ay* ratio in dystrophic dogs progressively increased in the thoracic region over the course of months, whereas that in the lumbar region increased slightly at the age of 10 and 11 months. The *Az* ratio in dystrophic dogs was slightly higher in the lumbar region compared with WT dogs at the age of 8 months. Attenuation of the *Ax* ratio in dystrophic dogs reflected a progressive decay in the forward propulsive force, whereas the increase in the *Ay* and *Az* ratios is indicative of a heightening motion for the medial-lateral and ventral-dorsal directions, respectively.

### Angular velocity parameters in dystrophic and WT dogs

**Fig 3:**
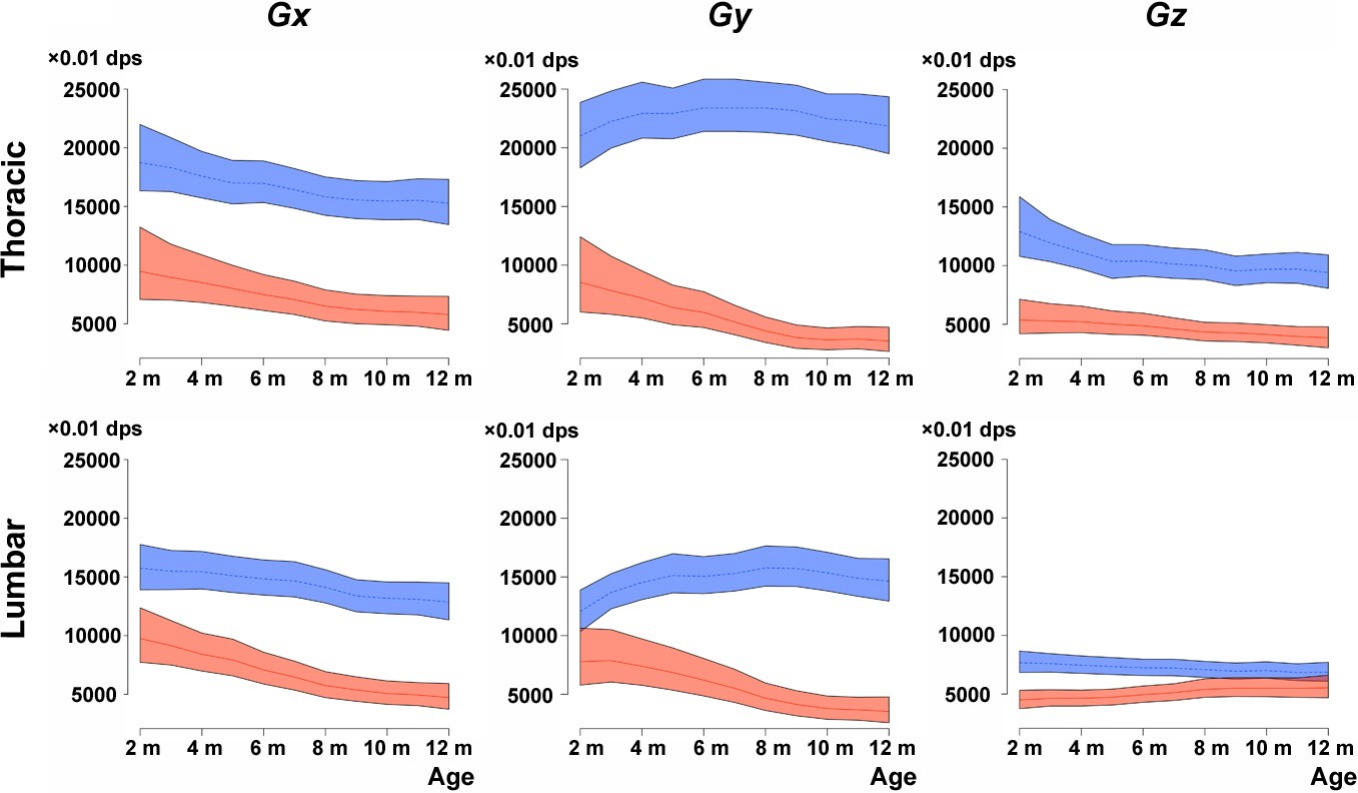
Angular velocity parameters of wild-type and dystrophic dogs at different ages. Averages of absolute values of angular velocity (*Gx*, *Gy*, and *Gz*). Lines and shaded surrounding areas in wild-type (WT, blue) and dystrophic dogs (red) indicate estimations and 95% credible intervals of the trends in angular velocity parameters, respectively.

Three-axial angular velocity of running for 15 m was examined in dystrophic and WT dogs. Angular velocity waves for the X, Y, and Z axes are shown in S2 Fig. All waves of dystrophic dogs were lower and had broader amplitudes than those of WT dogs during the gallop. These waves in dystrophic dogs were also lower during the trot than the gallop, but the Z-axial wave in the lumbar region was higher. The instantaneous vectors of angular velocity (*Gx*, *Gy*, *Gz*) were compared between dystrophic and WT dogs at different ages (Fig 3). Three-axial vectors of dystrophic dogs were lower than those of WT dogs, both in dorsal thoracic and lumbar regions, and were attenuated over the course of months, except for *Gz* in the lumbar region. *Gy* in the thoracic region in WT dogs largely increased and was drastically different from that in dystrophic dogs, even at the age of 2 months. *Gz* in the lumbar region in dystrophic dogs was lower compared with WT dogs, but had increased similarly to that in WT dogs at the age of 8 months.

### Relationship between multiple parameters of acceleration and angular velocity and clinical manifestations in dystrophic dogs

**Fig 4:**
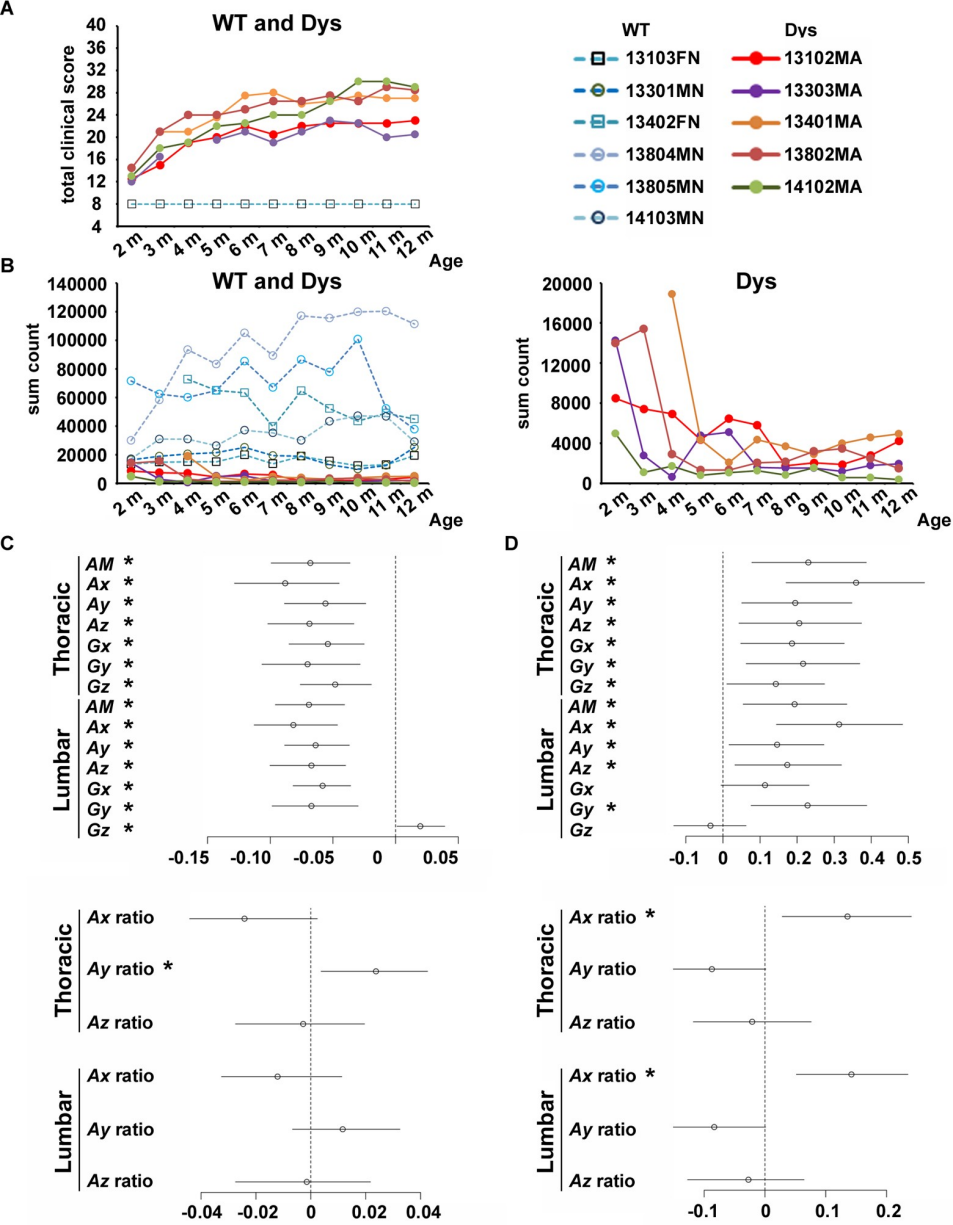
Relationship between multiple parameters and clinical evaluations in dystrophic dogs. (A) Total grading scores of clinical manifestation and (B) spontaneous locomotor activity at different ages. Circle and square markers indicate male and female dogs, respectively, in wild-type (WT) and dystrophic (Dys) dogs. Characteristics and subject IDs are described in Table 1. In panel (A), total grading scores of WT dogs are equal. In panels (B), all data of WT and dystrophic dogs are shown in left panel, and data of dystrophic dogs are shown in right panel. (C and D) Trend estimations between multiple parameters and clinical evaluations in dystrophic dogs. The circles indicate the point estimations of the coefficients of the clinical evaluations for each parameter (i.e., *AM*, *Ax*, *Ay*, etc.). The bars indicate the corresponding 95% credible intervals. Regarding the clinical evaluations, panel (C) shows total grading scores in clinical manifestations, and panel (D) shows spontaneous locomotor activity. *The credible intervals do not contain 0. This means that we can conclude with 95% confidence that the clinical evaluations have a positive (or negative) impact on the multiple parameters.

Multiple parameters of acceleration and angular velocity were examined regarding their relationship with clinical manifestations in dystrophic dogs. Total grading scores for clinical manifestations are shown over the course of months (Fig 4A). Total grading scores of all the dystrophic dogs were high at the age of 2 months and increased to various peak values between the age of 7 and 11 months, indicative of the variation in phenotypic severity. For trend estimation, the relationship between each parameter and the total grading score was represented as a coefficient (Fig 4C). The coefficients of the total grading scores for multiple parameters were less than 0, except for *Gz* in the lumbar region. These results revealed that multiple parameters mostly decreased with an exacerbation in severity, whereas *Gz* in the lumbar region increased. Among acceleration ratios, the coefficient for the *Ay* ratio in the thoracic region was greater than 0, indicating that acceleration in the medial-lateral direction in the thoracic region increased with exacerbation in severity.

### Relationship between multiple parameters of acceleration and angular velocity and spontaneous locomotor activity in dystrophic dogs

Next, we examined the relationship between multiple parameters of acceleration and angular velocity and spontaneous locomotor activity in the dystrophic dogs. Spontaneous locomotor activity is shown over the course of months (Fig 4B). Spontaneous locomotor activity in dystrophic dogs was variously decreased between the age of 2 and 5 months, and was lower that of WT dogs. For trend estimation, the coefficients of spontaneous locomotor activity for multiple parameters were greater than 0, except for *Gz* in the lumbar region (Fig 4D). An increase in multiple parameters was highly concomitant with spontaneous locomotor activity. Among acceleration ratios, the coefficients for *Ax* ratios in the dorsal thoracic and lumbar regions were also greater than 0. In contrast, *Ay* ratios in the dorsal thoracic and lumbar regions tended to be less than 0, but the credible intervals included 0.

### Correlation between *AM*s and muscle involvement in dystrophic dogs

**Fig 5:**
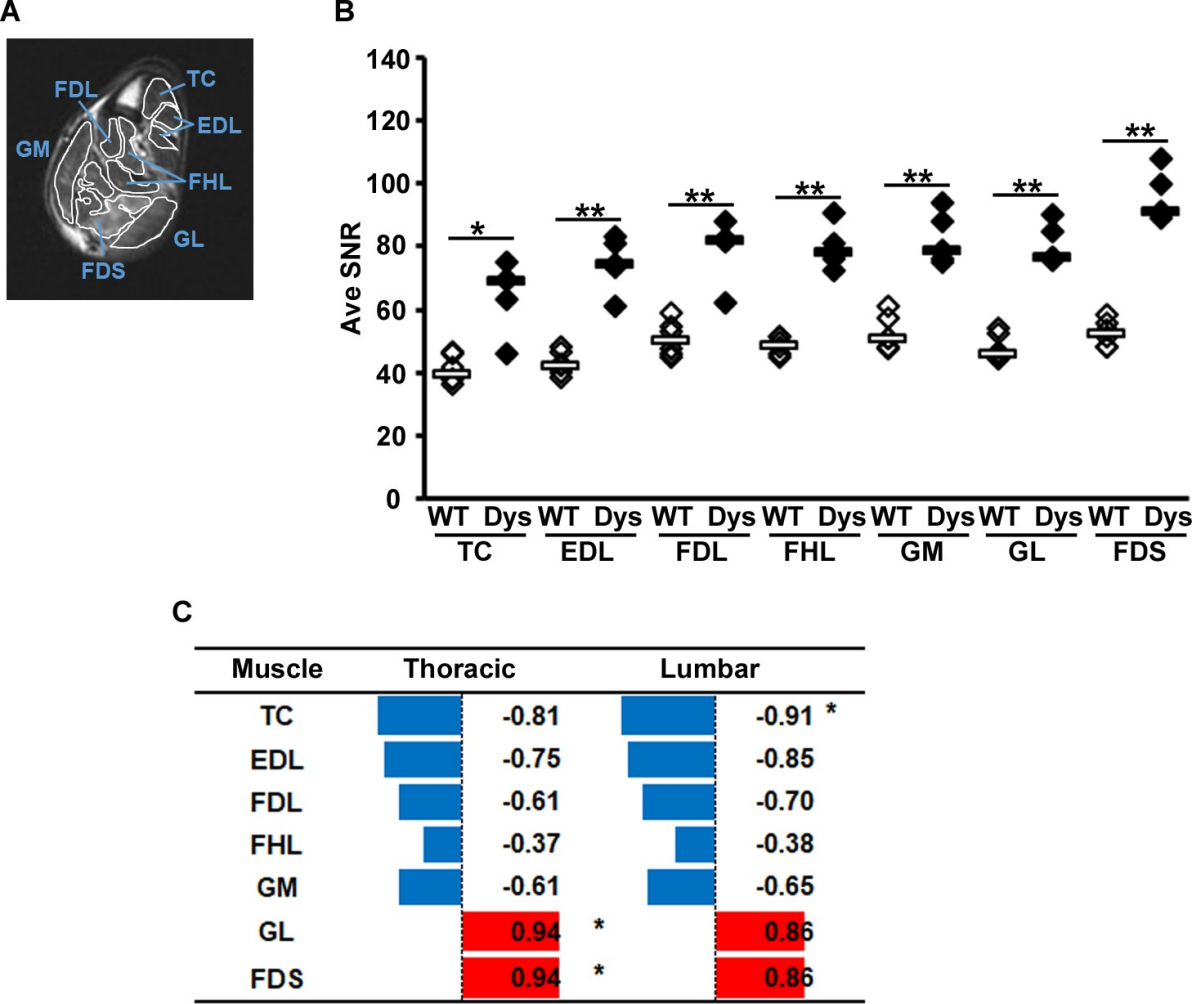
Correlation between acceleration magnitudes and muscle involvement in dystrophic dogs at the age of 1 year. Muscle involvement in crus muscles was evaluated with magnetic resonance imaging. (A) T2-weighted image of a transverse section of a lower limb of a dystrophic dog (13102MA). (B) Average signal-to-noise ratios (Ave SNRs) of crus muscles in T2-weighted images were compared between wild-type (WT, white diamonds) and dystrophic (Dys; black diamonds) dogs. (C) Pearson’s correlation coefficients between acceleration magnitudes and Ave SNRs of crus muscles of dystrophic dogs. Blue and red bars indicate negative and positive correlations, respectively. *P < 0.05. TC, tibialis cranialis; EDL, extensor digitorum longus; FDL, flexor digitorum longus; FHL, flexor hallucis longus; GM, gastrocnemius medial head; GL, gastrocnemius lateral head; FDS, flexor digitorum superficialis. Scatter plots for these data are shown in S3 Fig.

We analyzed correlation coefficients between *AM*s and MRI values in dystrophic dogs at the age of 1 year. In T2-weighted images, intense signal depicting muscle involvement was observed in the crus muscles of dystrophic dogs, and Ave SNRs of the crus muscles were significantly higher in dystrophic dogs compared with those in WT dogs (Fig 5A and 5B). Ave SNRs of crus muscles were compared with the correlation coefficient for *AM*s in the dorsal thoracic and lumbar regions in dystrophic dogs (Fig 5C). The overall pattern indicated that among fast muscles, Ave SNRs of the tibialis cranialis and extensor digitorum longus tended to be negatively correlated with *AM*s in the thoracic and lumbar regions, whereas among slow muscles, those of the gastrocnemius lateral head and flexor digitorum superficialis tended to be positively correlated with *AM*s in these regions, except for the gastrocnemius medial head, which showed a negative correlation. A significant correlation was observed between *AM* in the lumbar region and the Ave SNR of the tibialis cranialis and between *AM* in the thoracic region and Ave SNRs of the gastrocnemius lateral head and flexor digitorum superficialis. All correlations are also shown in scatter plots (S3 Fig). The correlation coefficients between other parameters of acceleration and angular velocity and Ave SNRs of crus muscles in dystrophic dogs are shown in S2 Table.

## Discussion

In the present study, we evaluated the gait in dystrophic dogs by measuring three-axial acceleration and angular velocity in the dorsal thoracic and lumbar regions and followed the dogs over the course of months. Multiple parameters during the disease course declined differently between the thoracic and lumbar regions. These parameters also accordingly decreased with exacerbation of clinical manifestations and a decrease in spontaneous locomotor activity. We also compared average SNRs on T2-weighted imaging and *AM*s. Acceleration and angular velocity would be novel outcome measures that reflect disease progression in hereditary muscle diseases.

In previous studies in CXMD_J_, we performed motor function tests including running 15 m down a hallway, recording the time from lateral recumbency to a standing position, and spontaneous locomotor activity [33, 34, 37]. Gait function in GRMD was recently evaluated with an accelerometer placed over the sternum, and a decline in the total power summed from three-axial acceleration was observed over the disease course [28, 29]. We found differences in acceleration between the dorsal thoracic and lumbar regions, and also reported the efficiencies of angular velocity in gait analysis of dystrophic dogs. From the present and previous studies, accelerometry provides quantitative, multifaceted kinematic indices in combination with conventional motor function tests.

### Kinematic features of acceleration in dystrophic dogs

*AM*s in the dorsal thoracic and lumbar regions in WT dogs increased, with peaks at the ages of 4 and 7–8 months, respectively. Thoracic and lumbar motion is strongly influenced by forelimb and hindlimb gait movement, respectively [44]. The load in the vertical direction in the standing position is applied to the forelimb greater than to the hindlimb with at least a ratio of 6:4 [44–48], suggesting that the forelimb is mainly loaded from the early stage when walking starts. In dystrophic dogs, acceleration, especially *Ax* and *AM* in the thoracic region, were prominently lower than those in WT dogs, even at the age of 2 months, and gradually decreased. However, those values in the lumbar region were slightly lower at the age of 2 months and drastically declined over the course of months. Thus, dystrophic dogs may experience muscle involvement, especially in the forelimb, due to the strut load before the onset of muscle weakness, leading to a decline in acceleration in the thoracic region. In previous studies in GRMD, forelimb muscles tend to be injured more severely than hindlimb muscles in the early phase including the neonatal stage, when groveling locomotion is observed, and at the age of 6–8 weeks, when standing on the extremities is observed [49, 50].

In CXMD_J_, a change in the gait pattern, such as from a gallop to a trot or walk, was observed in individual dogs over the course of months, reflecting the decline in acceleration indices, as previously reported in GRMD [28, 29]. Electromyographic activity of the hindlimbs in dogs is elevated more than that of the forelimbs during a gallop, and decreases during a trot or walk [47]. The gallop may become abnormal following hindlimb dysfunction in dystrophic dogs, resulting from hindlimb muscle involvement. The drastic decline in acceleration indices in the lumbar region is probably related to progressive involvement of hindlimb muscles during the disease course.

### Correlation between muscle involvement and acceleration

We performed MRI to detect involvement of hindlimb muscles and analyzed the correlation with acceleration, which reflects the gait of dystrophic dogs. All average SNRs of dystrophic crus muscles on T2-weighted images were significantly higher compared with those of WT dogs at the age of 1 year. Fan et al. reported that T2-weighted signals of thigh muscles of GRMD show higher values than those of WT dogs from the age of 3 to 9–12 months, depending on the muscle group; however, these signals decreased over the course of several months [51]. We observed that the average SNRs and *AM*s in fast muscles, especially tibialis cranialis and extensor digitorum longus, tended to show a negative correlation, indicating that individuals with worse muscle involvement showed lower acceleration. In previous studies in CXMD_J_ and GRMD, fast myofibers were prominently reduced from the age of 15 days in the early phase, whereas the population of slow myofibers increased according to myofiber type [26, 27]. Fast muscles in dystrophic dogs are also relatively highly injured in the early phase [49, 50]. Thus, involvement of fast muscles in dystrophic dogs may have already progressed at the age of 1 year, implying that the remaining, poorly functioning fast myofibers led to the lower values of *AM*s in gait.

On the other hand, average SNRs and *AM*s in slow muscles, especially the gastrocnemius lateral head and flexor digitorum superficialis, tended to show a positive correlation, indicating that individuals with worse muscle involvement showed higher acceleration, except for the gastrocnemius medial head, which showed a negative correlation. These correlations in slow muscles were not wholly determinate. Therefore, further studies are necessary to investigate the disease course over time in an increased number of dogs. Because multiple factors including cardiopulmonary dysfunction may potentially interfere with gait, simplifying the pathological conditions using correlation analysis is difficult. However, the present approach provides new findings that link muscle involvement with motor function tests.

### Kinematic features of angular velocity in dystrophic dogs

Angular velocity is useful for evaluating pathological conditions because it serves as an index of rotational movement of a directional axis [16]. We reported a change in angular velocity in gait analysis of dystrophic dogs for the first time. *Gy* in the thoracic region in WT dogs showed the highest value among angular velocity indices and notably increased over the course of months. Limb behavior during a gait in dogs causes a wider sweeping motion of the thorax by operating forelimb movement in the horizontal and vertical axes [44]. An increase in *Gy* in the thoracic region in WT dogs reflects dynamic forelimb motion during a gallop, and therefore, greater power is imposed on that leg. In dystrophic dogs, lower values were obtained compared with WT dogs, even at the age of 2 months, and largely declined over the course of months. The intense motion of the thorax causes muscle involvement of the forelimb by providing a strong load in the early phase in dystrophic dogs, leading to gait dysfunction and a reduced *Gy* in the thoracic region. These results are consistent with those of the *AM* in the thoracic region in dystrophic dogs. *Gz* in the lumbar region in WT dogs slightly decreased over time, whereas that in dystrophic dogs increased from the age of 2 months. Dystrophic dogs show the symptoms of ankylosis and supinated forelimbs due to muscle weakness from the age of 4 months, resulting in a waddling-like gait [28, 32]. The increase in *Gz* in the lumbar region in dystrophic dogs may be the result of waddling at the girdle. This result suggests the importance of motion evaluation in the lumbar region. Angular velocity is a potentially important measurement in gait analysis of hereditary neuromuscular disorders including DMD.

### Clinical application of acceleration and angular velocity

Multiple parameters of acceleration and angular velocity decreased according to disease severity as determined with clinical scales in dystrophic dogs. At the age of 2 months, which is the onset of muscle weakness in dystrophic dogs, clinical scales were only slightly increased, whereas the decline in *AM* in the thoracic region was more pronounced, suggesting that the *AM* index is remarkably sensitive for evaluation of the pathological condition. Gait dysfunction was also first reported to relate to spontaneous locomotor activity in dystrophic dogs, supporting the previous reports in DMD patients [14, 15, 18]. Accelerometry may be applicable in dystrophic dogs to investigate a decrease in motivation for daily activity due to motor dysfunction.

### Conclusion

Acceleration and angular velocity are efficient outcome measures for quantification of motor function according to the disease course and severity. These parameters should be widely used in motor function tests in patients with hereditary neuromuscular disorders including DMD. These parameters may be also useful for investigation of the relationship between motor function and muscle involvement of fast and slow muscles, leading to elucidation of the dystrophic pathology.

## Supporting information

S1 Fig Acceleration waves. Acceleration waves for the X, Y, and Z axes during a gallop in wild-type (WT) (13103FN, left) and dystrophic (13102MA, middle) dogs and a trot in a dystrophic dog (13401MA, right) at the age of 8 months. All time scales are for 2 seconds. Subject IDs and characteristics are described in Table 1.
(TIF)

S2 Fig Angular velocity waves. Angular velocity waves for the X, Y, and Z axes during a gallop in wild-type (13103FN, left) and dystrophic (13102MA, middle) dogs and a trot in a dystrophic dog (13401MA, right) at the age of 8 months. All time scales are shown for 2 seconds. Angular velocity waves were acquired concomitantly with the acceleration waves shown in S1 Fig. Subject IDs and characteristics are described in Table 1.
(TIF)

S3 Fig Scatter plots comparing acceleration magnitude (*AM*) and the average signal-to-noise ratio (Ave SNR) of crus muscles on T2-weighted images. All data were derived from dystrophic dogs at the age of 1 year. TC, tibialis cranialis; EDL, extensor digitorum longus; FDL, flexor digitorum longus; FHL, flexor hallucis longus; GM, gastrocnemius medial head; GL, gastrocnemius lateral head; FDS, flexor digitorum superficialis.
(TIF)

S1 Table Ambulatory patterns of dystrophic dogs at different ages. (DOCX)

S2 Table Pearsons's correlation coefficients between multiple parameters and average signal-to-noise ratios of crus muscles in dystrophic dogs. (DOCX)
