# Supplementary figures and images for: Accelerometric outcomes of motor function related to clinical evaluations and muscle involvement in dystrophic dogs
Source: PLoS One. 2018 Dec 11;13(12):e0208415. doi: 10.1371/journal.pone.0208415 (PMC6289438; doi:10.1371/journal.pone.0208415)

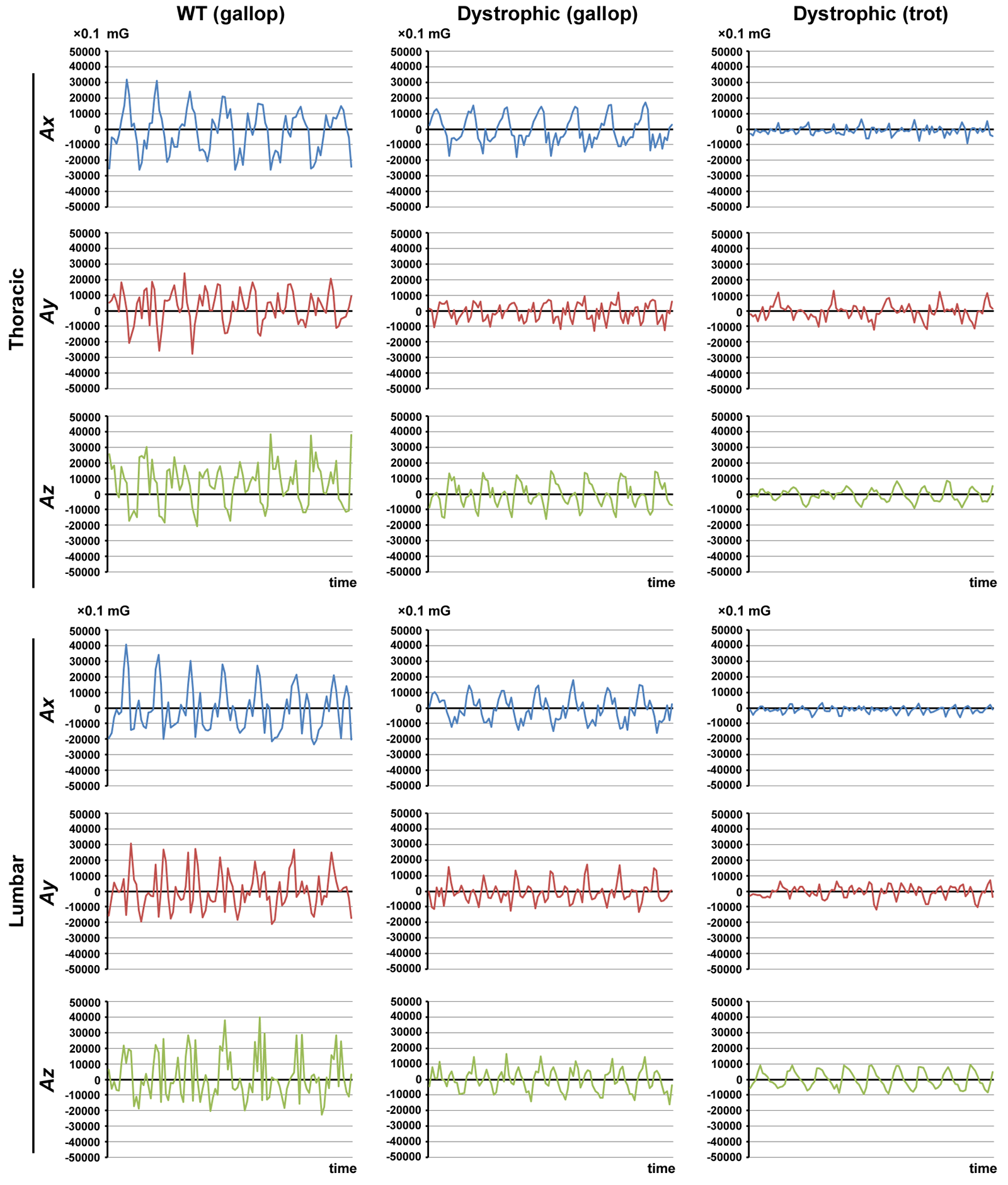

Supplement: S1 Fig — Acceleration waves for the X, Y, and Z axes during a gallop in wild-type (WT) (13103FN, left) and dystrophic (13102MA, middle) dogs and a trot in a dystrophic dog (13401MA, right) at the age of 8 months. All time scales are for 2 seconds. Subject IDs and characteristics are described in Table 1. (TIF) [file pone.0208415.s001.tif]

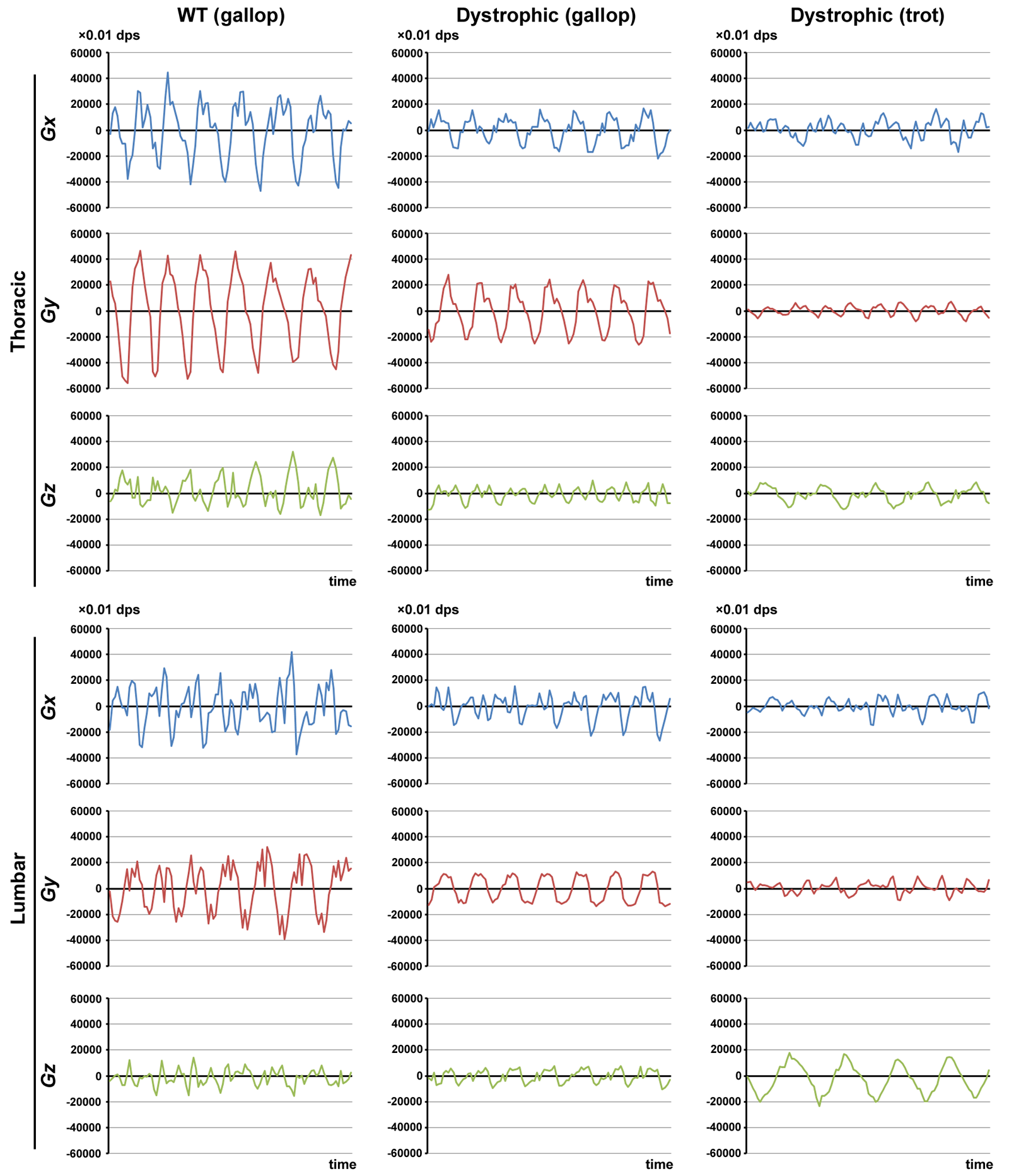

Supplement: S2 Fig — Angular velocity waves for the X, Y, and Z axes during a gallop in wild-type (13103FN, left) and dystrophic (13102MA, middle) dogs and a trot in a dystrophic dog (13401MA, right) at the age of 8 months. All time scales are shown for 2 seconds. Angular velocity waves were acquired concomitantly with the acceleration waves shown in S1 Fig. Subject IDs and characteristics are described in Table 1. (TIF) [file pone.0208415.s002.tif]

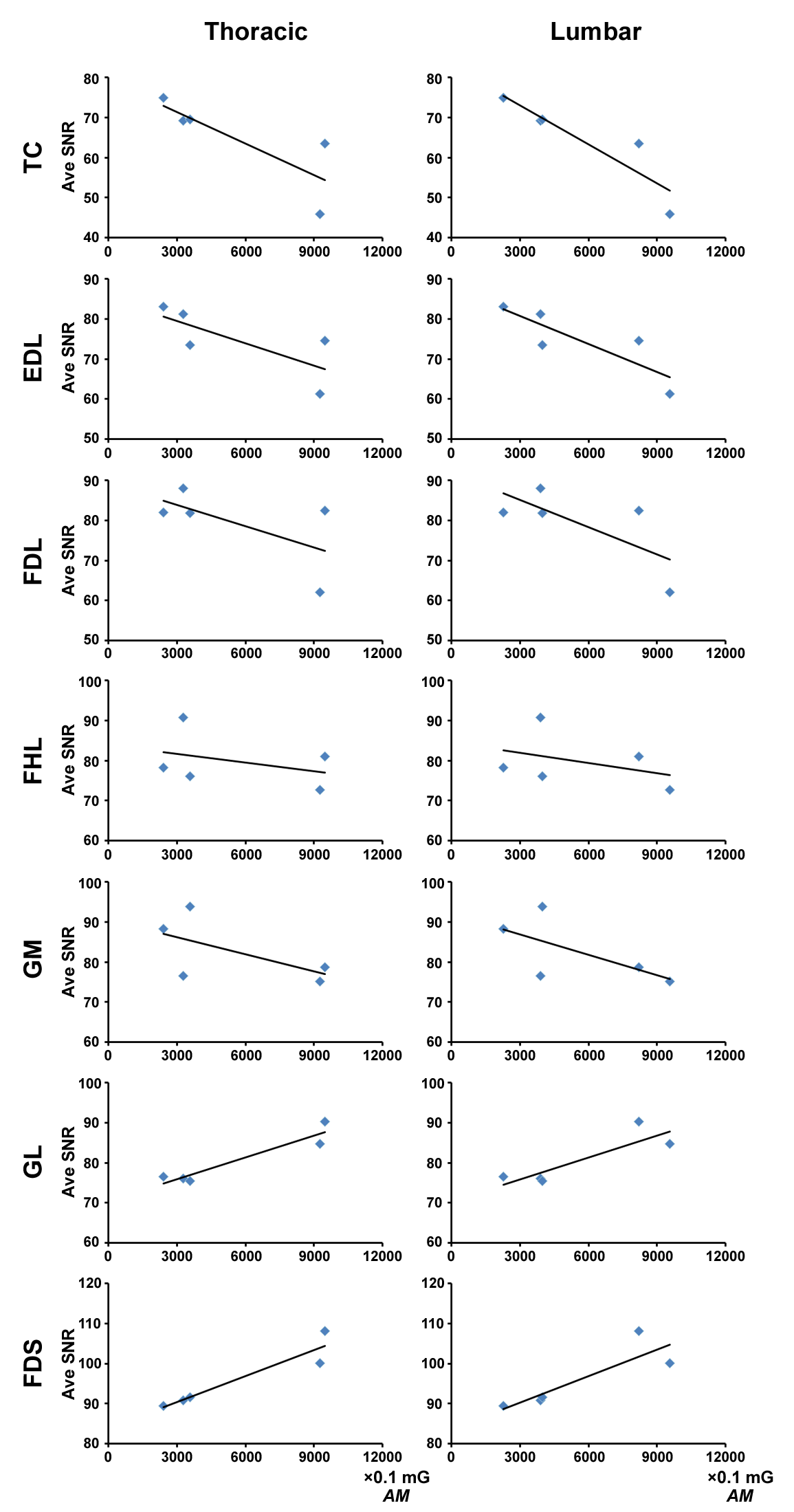

Supplement: S3 Fig — All data were derived from dystrophic dogs at the age of 1 year. TC, tibialis cranialis; EDL, extensor digitorum longus; FDL, flexor digitorum longus; FHL, flexor hallucis longus; GM, gastrocnemius medial head; GL, gastrocnemius lateral head; FDS, flexor digitorum superficialis. (TIF) [file pone.0208415.s003.tif]
