# Supplementary material for: Accelerometric outcomes of motor function related to clinical evaluations and muscle involvement in dystrophic dogs
Source: PLoS One. 2018 Dec 11;13(12):e0208415. doi: 10.1371/journal.pone.0208415 (PMC6289438; doi:10.1371/journal.pone.0208415)
Supplement: S1 Table — (DOCX) [file pone.0208415.s004.docx]

S1 Table. Ambulatory patterns of dystrophic dogs at different ages.

| Age | Trial | Subject ID | | | | |
| --- | --- | --- | --- | --- | --- | --- |
|  |  | 13102MA | 13303MA | 13401MA | 13802MA | 14102MA |
| 2 m | 1st | gallop | gallop | gallop | gallop | trot |
|  | 2nd | gallop | gallop | gallop | gallop | gallop |
|  | 3rd | gallop | gallop | gallop | gallop | gallop |
|  | 4th | gallop | gallop | gallop | gallop | gallop |
| 3 m | 1st | gallop | gallop | gallop | gallop | trot |
|  | 2nd | gallop | gallop | gallop | gallop | trot |
|  | 3rd | gallop | gallop | gallop | gallop | trot |
|  | 4th | gallop | gallop | trot | gallop | trot |
| 4 m | 1st | gallop | gallop | gallop | gallop | trot, gallop |
|  | 2nd | gallop, trot | gallop | gallop | gallop | walk, trot, walk |
|  | 3rd | gallop | gallop | gallop | gallop | trot |
|  | 4th | gallop | gallop | gallop | gallop | walk, trot |
| 5 m | 1st | gallop | gallop | gallop | gallop | trot, walk |
|  | 2nd | gallop | gallop | gallop | gallop | trot |
|  | 3rd | gallop | gallop | gallop, trot | gallop | walk |
|  | 4th | gallop | gallop | trot | gallop | trot, walk |
| 6 m | 1st | gallop | gallop | gallop | gallop | gallop, walk |
|  | 2nd | gallop | gallop | gallop | gallop | walk |
|  | 3rd | gallop | gallop | gallop | gallop | trot |
|  | 4th | gallop | gallop, trot | trot | gallop | walk |
| 7 m | 1st | gallop, trot | gallop | gallop | gallop, walk | trot |
|  | 2nd | gallop, trot | gallop | trot | gallop | trot |
|  | 3rd | gallop, trot | gallop | gallop | gallop, walk | walk, trot |
|  | 4th | trot | gallop | trot | trot, walk | trot |
| 8 m | 1st | gallop | gallop | trot | gallop, walk | trot, walk |
|  | 2nd | gallop | trot | trot | trot | trot |
|  | 3rd | gallop | trot, walk | trot | gallop, walk, trot | walk, trot, walk |
|  | 4th | gallop | trot | trot | trot | trot |
| 9 m | 1st | gallop | gallop | trot | trot | walk |
|  | 2nd | gallop | gallop, walk | trot | trot | walk |
|  | 3rd | gallop | gallop | trot, walk | trot, walk | walk |
|  | 4th | gallop, trot | trot | trot, walk | trot, walk | walk |
| 10 m | 1st | gallop | walk | trot | gallop, walk | walk |
|  | 2nd | trot | gallop | trot | walk | walk |
|  | 3rd | trot | trot, walk | trot | gallop, trot | walk |
|  | 4th | trot | trot, walk | trot | walk | walk |
| 11 m | 1st | gallop, trot | gallop | trot | gallop, walk | walk |
|  | 2nd | gallop | gallop | gallop, trot | walk, trot | walk |
|  | 3rd | trot | gallop, walk | gallop | gallop, walk | failure |
|  | 4th | trot | gallop | gallop | walk | failure |
| 12 m | 1st | trot, gallop | gallop, walk | gallop | trot, walk | walk |
|  | 2nd | trot | trot, walk | trot | walk | walk |
|  | 3rd | trot | trot, walk | trot | walk | walk |
|  | 4th | trot | trot, walk | gallop | walk | walk |

Ambulatory patterns are shown in order of four trials at the ages of 2-12 months (m). Subject ID 14102MA at the age of 11 m did not complete the 3rd trial, so the 3rd and 4th trials are treated as failures.
