# Supplementary material for: Accelerometric outcomes of motor function related to clinical evaluations and muscle involvement in dystrophic dogs
Source: PLoS One. 2018 Dec 11;13(12):e0208415. doi: 10.1371/journal.pone.0208415 (PMC6289438; doi:10.1371/journal.pone.0208415)
Supplement: S2 Table — (DOCX) [file pone.0208415.s005.docx]

S2 Table. Pearsons's correlation coefficients between multiple parameters and average signal-to-noise ratios of crus muscles in dystrophic dogs.

|  |  | TC |  | EDL |  | FDL |  | FHL |  | GM |  | GL |  | FDS |  |
| --- | --- | --- | --- | --- | --- | --- | --- | --- | --- | --- | --- | --- | --- | --- | --- |
| Thoracic | *AM* | -0.810 |  | -0.750 |  | -0.611 |  | -0.365 |  | -0.606 |  | 0.944 | * | 0.943 | * |
|  | *Ax* | -0.727 |  | -0.676 |  | -0.516 |  | -0.325 |  | -0.560 |  | 0.971 | ** | 0.978 | ** |
|  | *Ay* | -0.772 |  | -0.695 |  | -0.563 |  | -0.316 |  | -0.627 |  | 0.966 | ** | 0.960 | ** |
|  | *Az* | -0.897 | * | -0.850 |  | -0.729 |  | -0.451 |  | -0.599 |  | 0.875 |  | 0.871 |  |
|  | *Ax* ratio | 0.582 |  | 0.480 |  | 0.206 |  | -0.315 |  | 0.688 |  | -0.239 |  | -0.311 |  |
|  | *Ay* ratio | 0.685 |  | 0.870 |  | 0.814 |  | 0.823 |  | -0.120 |  | -0.093 |  | -0.119 |  |
|  | *Az* ratio | -0.869 |  | -0.975 | ** | -0.865 |  | -0.690 |  | -0.192 |  | 0.311 |  | 0.339 |  |
|  | *Gx* | -0.408 |  | -0.322 |  | -0.177 |  | -0.086 |  | -0.495 |  | 0.974 | ** | 0.975 | ** |
|  | *Gy* | -0.501 |  | -0.440 |  | -0.274 |  | -0.176 |  | -0.489 |  | 0.987 | ** | 0.996 | ** |
|  | *Gz* | -0.992 | ** | -0.974 | ** | -0.892 | * | -0.532 |  | -0.539 |  | 0.555 |  | 0.552 |  |
| Lumbar | *AM* | -0.906 | * | -0.846 |  | -0.703 |  | -0.381 |  | -0.649 |  | 0.859 |  | 0.862 |  |
|  | *Ax* | -0.765 |  | -0.710 |  | -0.529 |  | -0.296 |  | -0.599 |  | 0.949 | * | 0.963 | ** |
|  | *Ay* | -0.780 |  | -0.700 |  | -0.524 |  | -0.237 |  | -0.666 |  | 0.941 |  | 0.951 | * |
|  | *Az* | -0.987 | * | -0.951 | * | -0.859 |  | -0.507 |  | -0.591 |  | 0.684 |  | 0.678 |  |
|  | *Ax* ratio | 0.645 |  | 0.647 |  | 0.663 |  | 0.288 |  | 0.263 |  | 0.233 |  | 0.233 |  |
|  | *Ay* ratio | 0.910 | * | 0.995 | ** | 0.904 | * | 0.735 |  | 0.251 |  | -0.467 |  | -0.481 |  |
|  | *Az* ratio | -0.897 | * | -0.984 | ** | -0.872 |  | -0.662 |  | -0.253 |  | 0.349 |  | 0.374 |  |
|  | *Gx* | -0.503 |  | -0.413 |  | -0.200 |  | -0.016 |  | -0.591 |  | 0.957 | * | 0.981 | ** |
|  | *Gy* | -0.766 |  | -0.712 |  | -0.542 |  | -0.317 |  | -0.591 |  | 0.954 | * | 0.964 | ** |
|  | *Gz* | -0.544 |  | -0.546 |  | -0.547 |  | -0.596 |  | -0.245 |  | 0.896 | * | 0.855 |  |

*P < 0.05, **P < 0.01. TC, tibialis cranialis; EDL, extensor digitorum longus; FDL, flexor digitorum longus; FHL, flexor hallucis longus; GM, gastrocnemius medial head; GL, gastrocnemius lateral head; FDS, flexor digitorum superficialis.
